# Supplementary material for: Metabolic landscape of the tumor microenvironment at single cell resolution
Source: Nat Commun. 2019 Aug 21;10:3763. doi: 10.1038/s41467-019-11738-0 (PMC6704063; doi:10.1038/s41467-019-11738-0)
Supplement: Supplementary file 1 — Supplementary information [file 41467_2019_11738_MOESM1_ESM.pdf]

Supplementary Information for

Metabolic landscape of the tumor microenvironment at single cell resolution

Xiao et al.

Supplementary Methods

Comparison of t-SNE results using relative mutual information

The relative mutual information metric was used to evaluate whether two clustering results are statistically similar or different. Specifically, we use $C = \{C_1, \dots\dots, C_k\}$ to represent the t-SNE clustering results generated by using the expression profile of metabolic genes, randomly selected set of genes or the complete set of genes. For each clustering pattern the probability distribution P_C is defined as:

$$P_c(i) = \frac{n_i}{N} \qquad (1)$$

Where n_i is the number of samples in the cluster C_i and N is the total number of samples in dataset. For a pair of clustering results $P_{C1} = (p_1 \dots p_n)$ and $P_{C2} = (q_1 \dots q_n)$, the mutual information of each pair of clusters $I(p, q)$ is defined as:

$$I(p,q) = R(i,j) log \frac{R(i,j)}{P_{C_1}(i) P_{C_2}(j)} \qquad (2)$$

Where $R(i, j)$ is the joint probability distribution defined as:

$$R(i,j) = \frac{|C_{1,i} \cap C_{2,j}|}{N} \qquad (3)$$

In which $C_{1,i}$ and $C_{2,j}$ are the i-th and j-th cluster of C_1 and C_2, respectively. Let n_i and m_j denote the numbers of samples in $C_{1,i}$ and $C_{2,j}$. The mutual information between P_{C1} and P_{C2} is then defined as the sum of all $I(p,q)$,

$$I(P_{C_1}, P_{C_2}) = \sum I(p,q) = \sum_{i,j} R(i,j) log \frac{R(i,j)}{P_{C_1}(i) P_{C_2}(j)} = \sum_{i,j} \frac{|C_{1,i} \cap C_{2,j}|}{N} log \frac{N \cdot |C_{1,i} \cap C_{2,j}|}{n_i m_j} \qquad (4)$$

The relative mutual information of P_{C1} and P_{C2} is defined as:

$$I'(P_{C_1}, P_{C_2}) = \frac{I(P_{C_1}, P_{C_2})}{\text{Max}\{I(P_{C_1}, P_{C_2})\}} \qquad (5)$$

Where $Max\{I(P_{C1}, P_{C2})\}$ is the maximal possible mutual information between P_{C1} and P_{C2}. A value of $I'(P_{C1}, P_{C2})$ close to 1 means nearly perfect consistency between C_1 and C_2.

Supplementary Figure 1

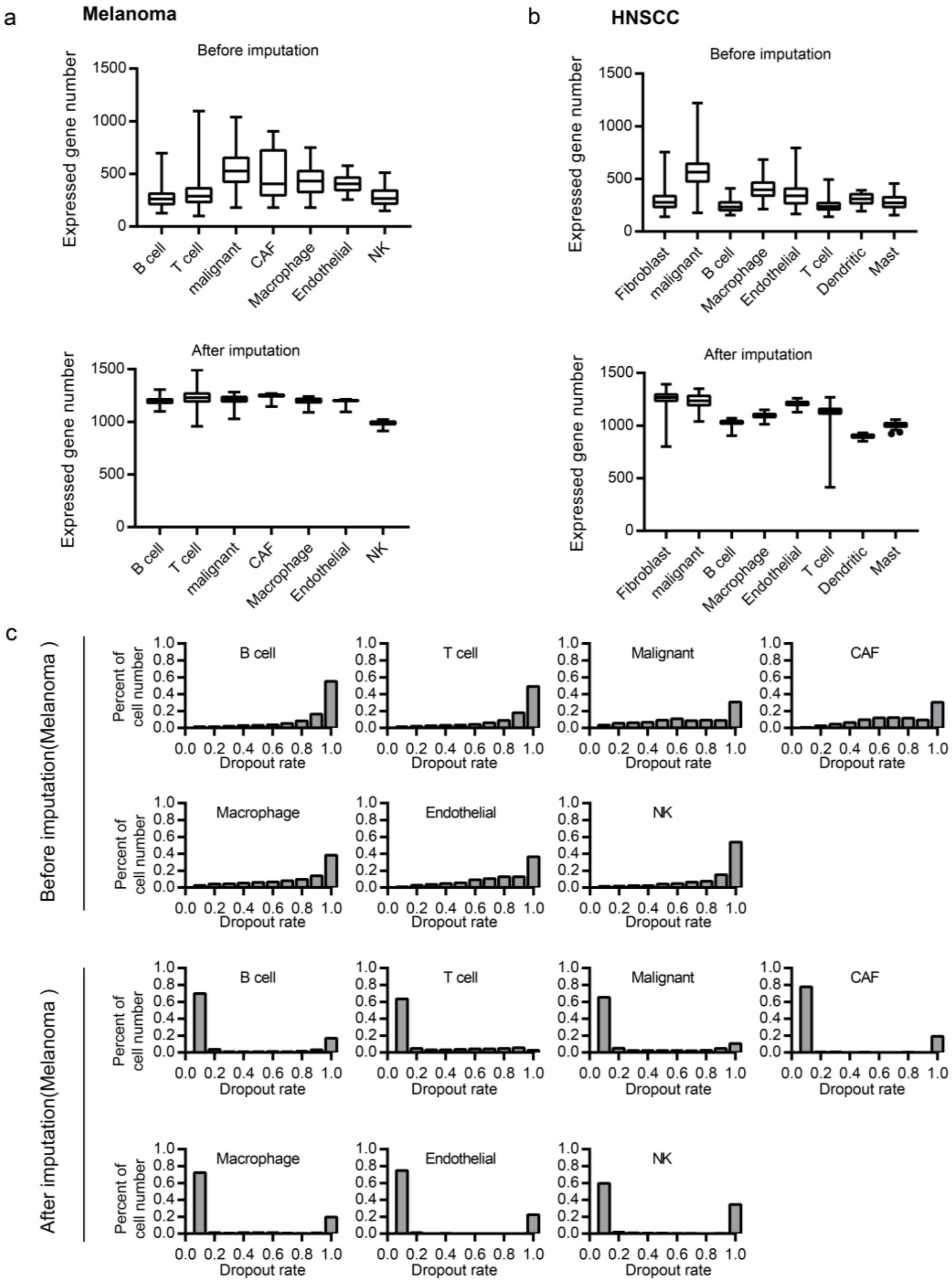

Supplementary Figure 1 (continued)

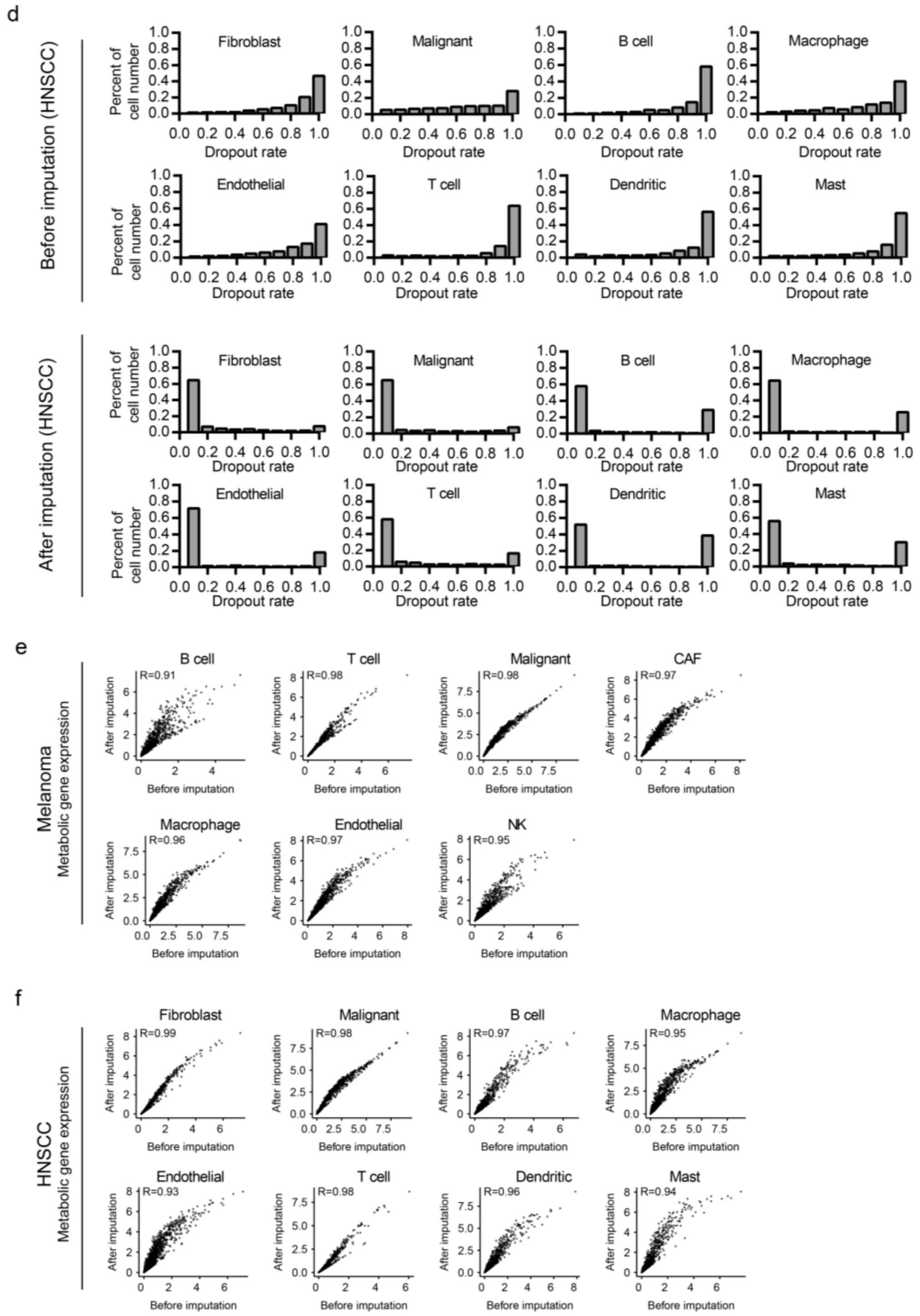

Supplementary Figure 1. (Related to Figure 1)

(a) Distributions of numbers of expressed genes (i.e. genes with non-zero expression value) before and after imputation in different cell types from the melanoma dataset. Box-plot elements: box limits, 25 and 75 percentiles; center line, median; whiskers, minimal and maximal values.

(b) Same as in (a) but for the squama cell carcinoma of head and neck (HNSCC) dataset.

(c) Distributions of drop-out rates (i.e. fraction of zero gene expression values) in different cell types before and after imputation in the melanoma dataset.

(d) Same as in (c) but for the HNSCC dataset.

(e) Scatter plots comparing average metabolic gene expression levels before and after imputation in different cell types from the melanoma dataset.

(f) Same as in (e) but for the HNSCC dataset.

Supplementary Figure 2

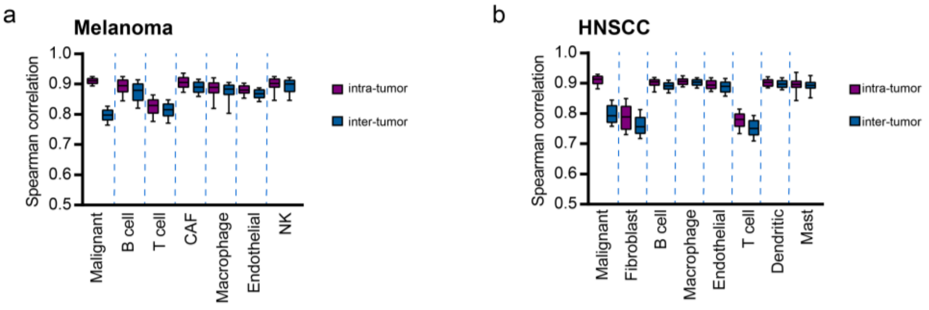

Supplementary Figure 2. (Related to Figure 1)

(a) Distributions of Spearman's rank correlation coefficients of metabolic gene expression between 500 randomly selected pairs of cells from the same tumor (i.e. intra-tumor) or from different tumors (i.e. inter-tumor) in the melanoma dataset. Box-plot elements: box limits, 25 and 75 percentiles; center line, median; whiskers, minimal and maximal values.

(b) Same as in (a) but for the HNSCC dataset.

Supplementary Figure 3

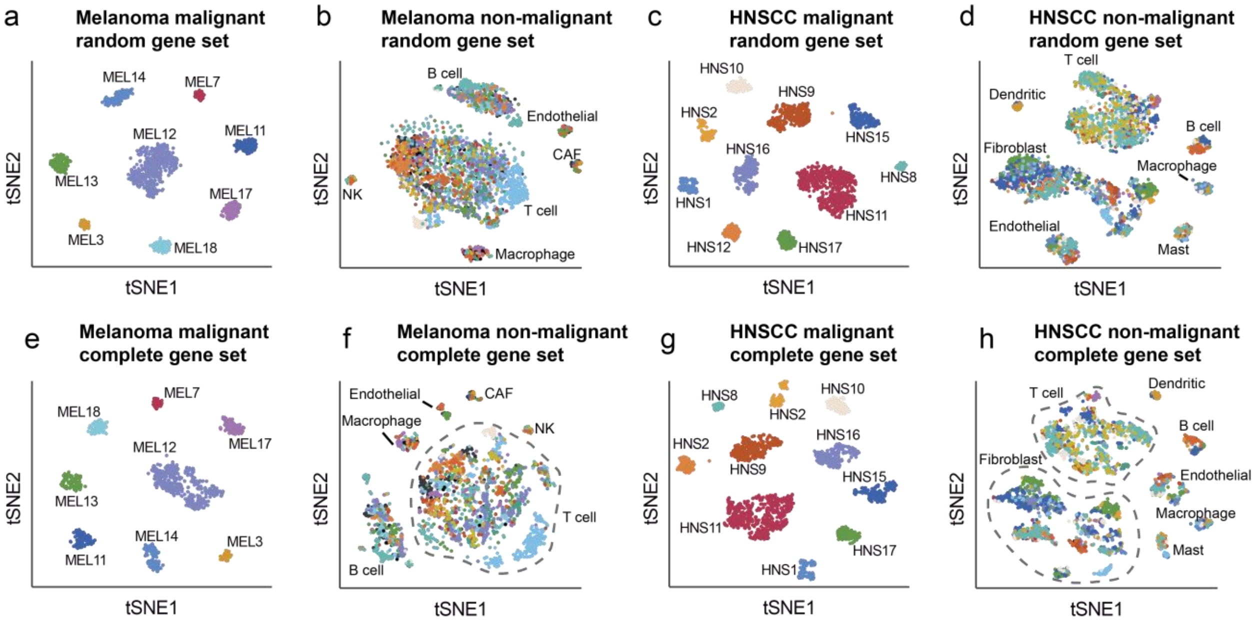

Supplementary Figure 3. (Related to Figure 1)

(a) t-SNE distribution of cells based on expression values of 1566 randomly selected genes in malignant cells from the melanoma dataset. The color of each dot indicates the tumor from which the cell was derived.

(b) same as in (a) but for non-malignant cells from the melanoma dataset.

(c) same as in (a) but for malignant cells from the HNSCC dataset.

(d) same as in (a) but for non-malignant cells from the HNSCC dataset.

(e) same as in (a) but based on expression values of the complete gene set.

(f) same as in (b) but based on expression values of the complete gene set.

(g) same as in (c) but based on expression values of the complete gene set.

(h) same as in (d) but based on expression values of the complete gene set.

Supplementary Figure 4

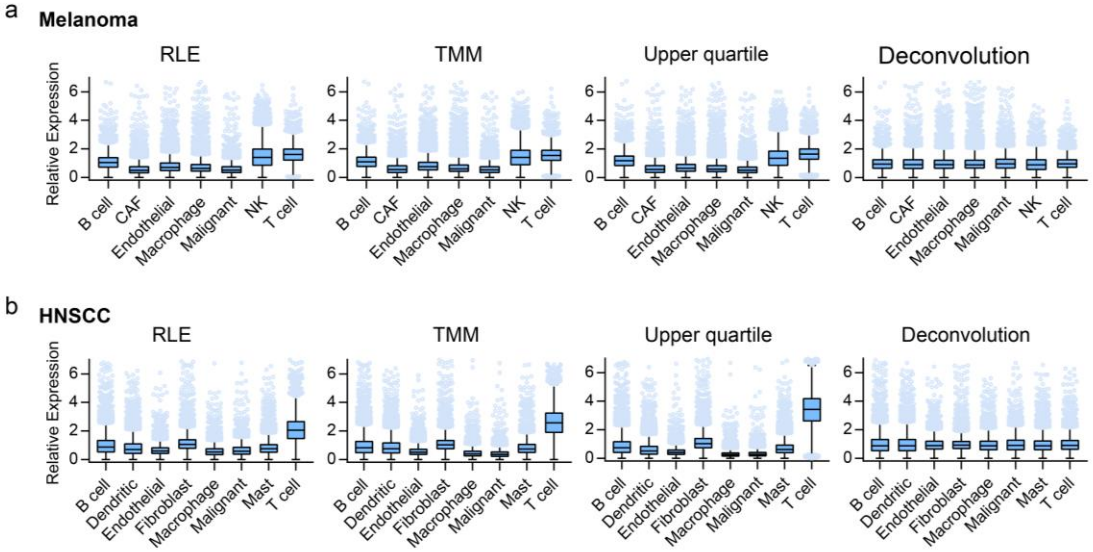

Supplementary Figure 4. (Related to Figure 2)

(a) Distributions of relative gene expression levels in cell types from the melanoma dataset after data normalization using relative log expression (RLE), trimmed mean of M-values (TMM), upper quartile or deconvolution method. Box-plot elements: box limits, 25 and 75 percentiles; center line, median; whiskers, 1.5 × interquartile range (IQR); points, outliers.

(b) Same as in (a) but for the HNSCC dataset.

Supplementary Figure 5

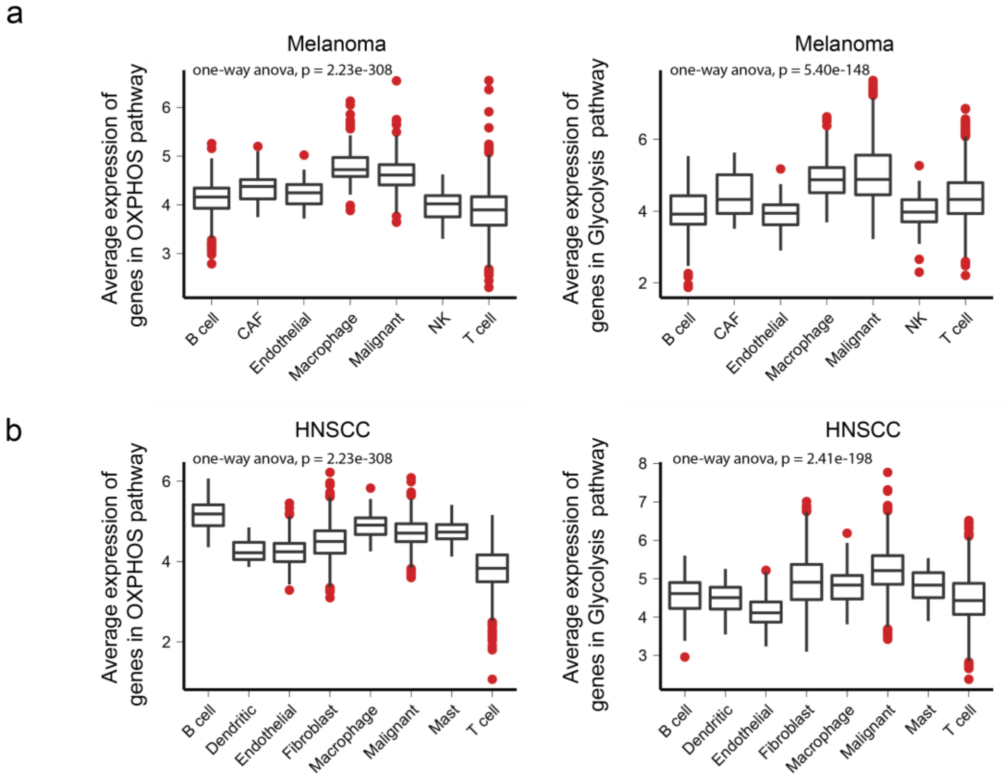

Supplementary Figure 5. (Related to Figure 2)

(a) Distributions of average OXPHOS (left) and glycolysis (right) gene expression levels in cell types in the melanoma dataset.

(b) Same as in (a) but for the HNSCC dataset.

P-values in each graph were computed using one-way ANOVA. Box-plot elements: box limits, 25 and 75 percentiles; center line, median; whiskers, 1.5 × interquartile range (IQR); points, outliers.

Supplementary Figure 6

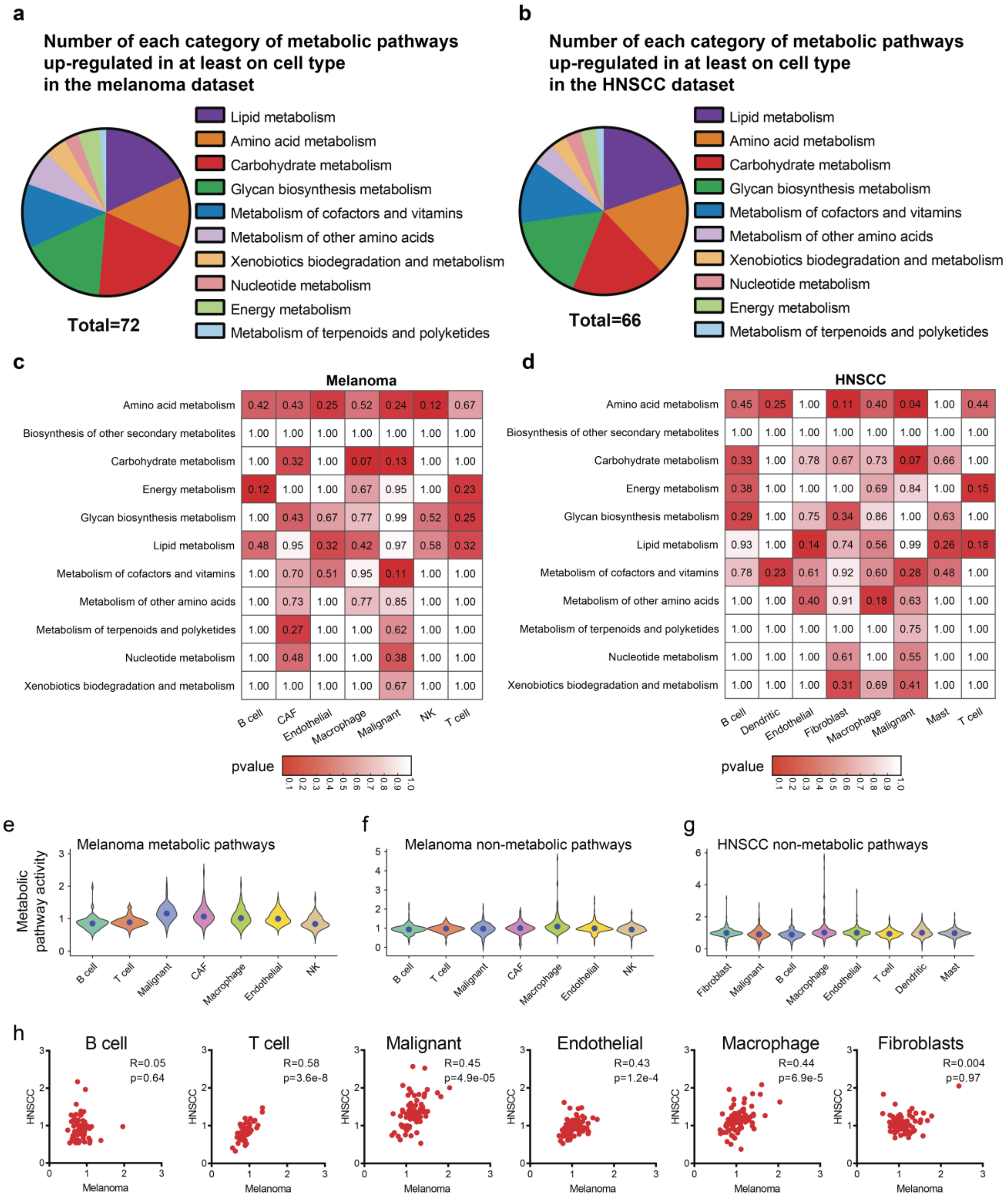

Supplementary Figure 6. (Related to Figure 2)

(a) Numbers of different categories of metabolic pathways up-regulated in at least one cell type in the melanoma dataset.

(b) Same as in (a) but for the HNSCC dataset.

(c) One-sided Fisher's exact test p-values for enrichment of categories of pathways in pathways up-regulated in different cell types in the melanoma dataset.

(d) Same as in (c) but for the HNSCC dataset.

(e) Distributions of metabolic pathway activity scores in cell types in the melanoma dataset.

(f) Distributions of non-metabolic pathway activity scores in cell types in the melanoma dataset.

(g) Same as in (f) but for the HNSCC dataset.

(h) Scatter plots comparing metabolic pathway activities between the melanoma and HNSCC datasets for cell types shared by the two datasets. The Spearman correlation coefficient is shown on the right of each graph

Supplementary Figure 7

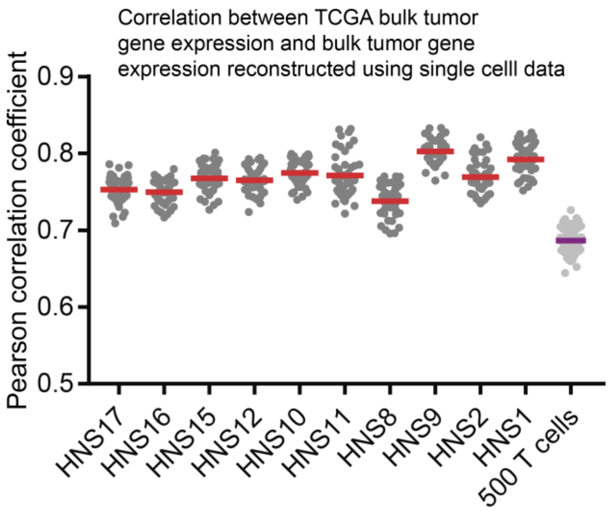

Supplementary Figure 7. (Related to Figure 2)

Distributions of Pearson correlation coefficients between TCGA bulk tumors and bulk tumors reconstructed using the single cell dataset. The horizontal line indicating the mean value.

Supplementary Figure 8

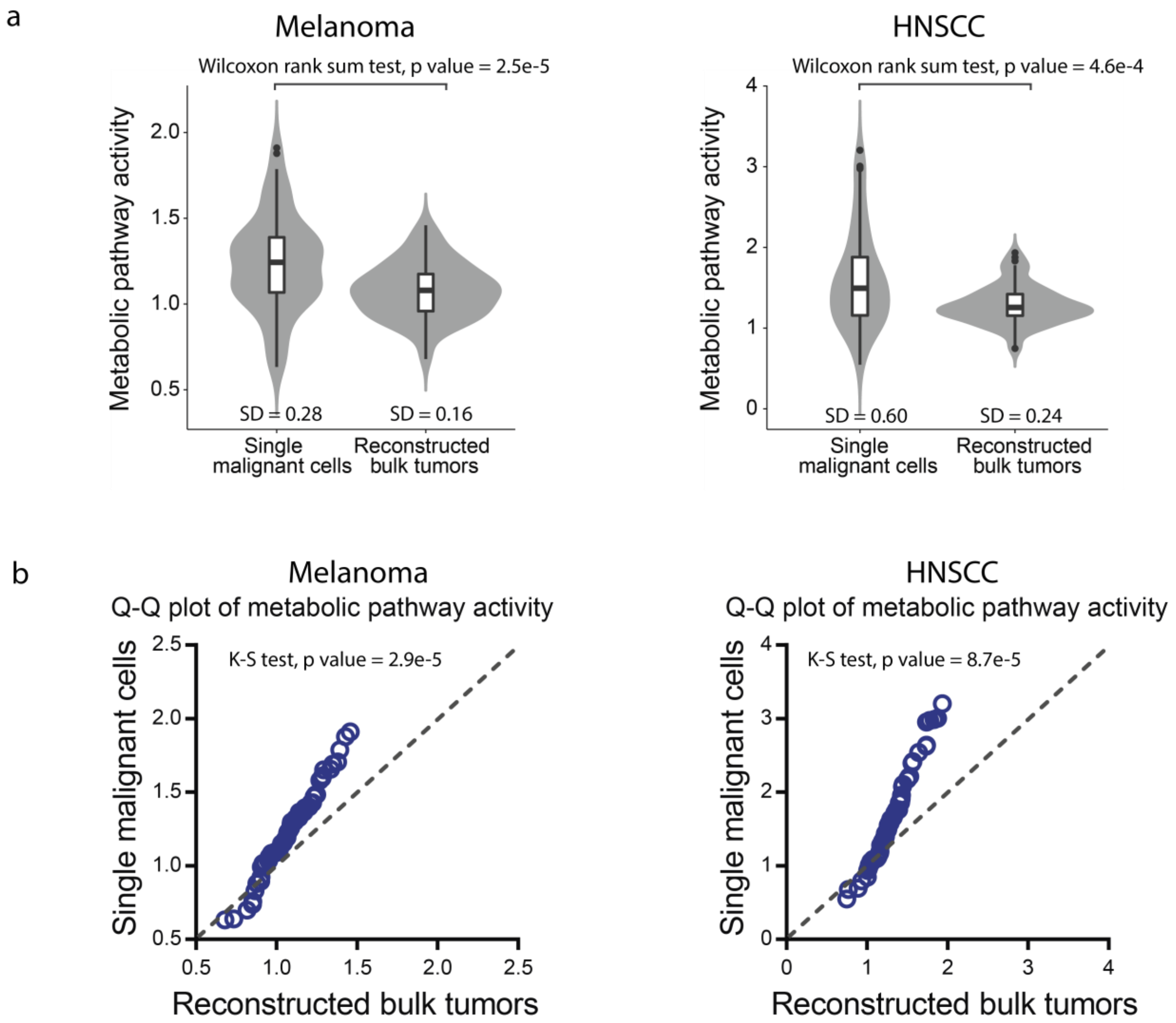

Supplementary Figure 8. (Related to Figure 2)

(a) Violin plots and box plots comparing the distributions of metabolic pathway activities in single malignant cells and reconstructed bulk tumors from the melanoma (left) and HNSCC (right) datasets. The standard variation (SD) values of metabolic pathway activities in each group are shown on the bottom. P-values were computed using one-sided Wilcoxon's rank-sum test. Box-plot elements: box limits, 25 and 75 percentiles; center line, median; whiskers, 1.5 × interquartile range (IQR); points, outliers.

(b) Quantile-quantile (Q-Q) plots showing the discrepancy between single malignant cells and reconstructed bulk tumors in distribution of metabolic pathway activities for the melanoma (left) and HNSCC (right) datasets. P-values were computed using one-sided Kolmogorov–Smirnov (K-S) test.

Supplementary Figure 9

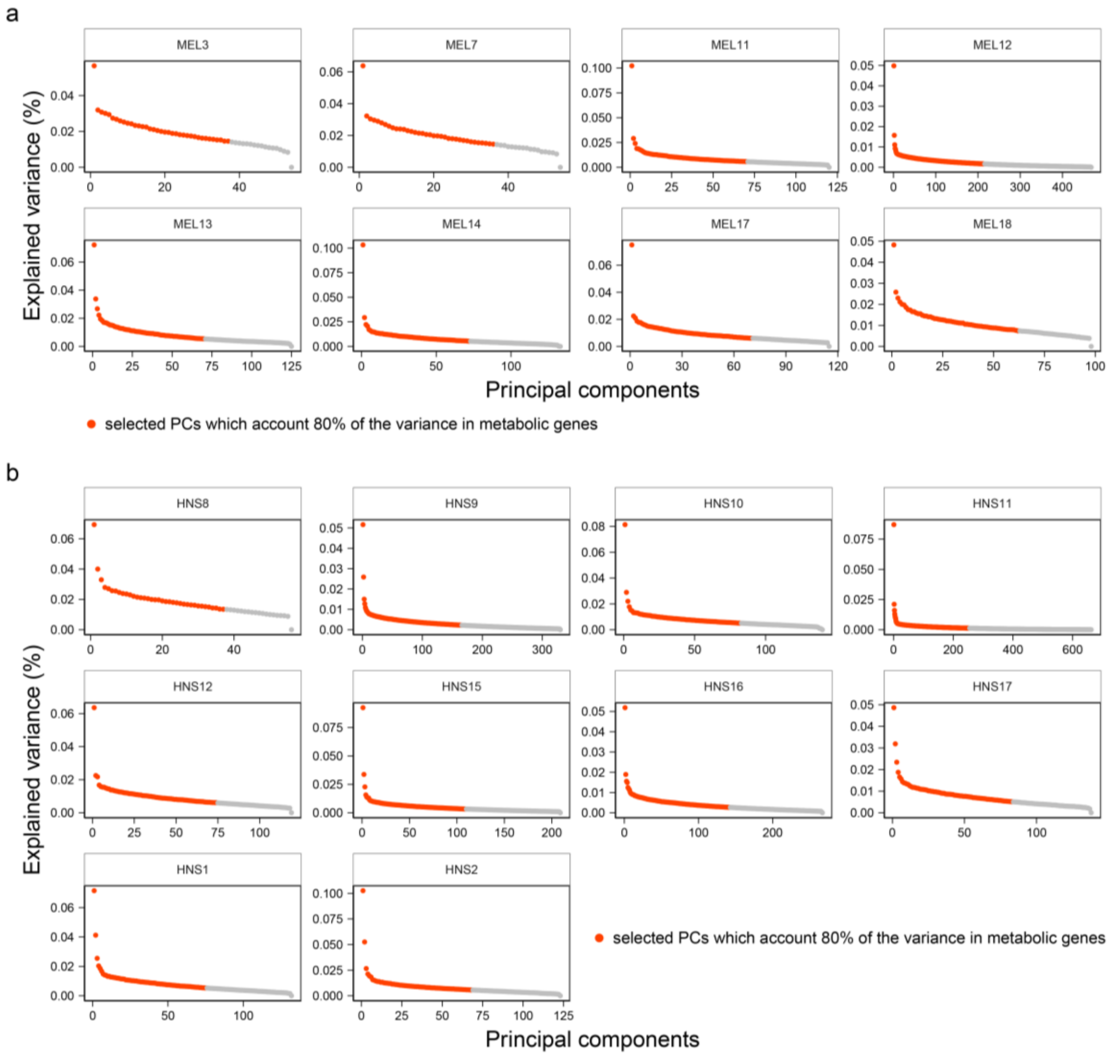

Supplementary Figure 9. (Related to Figure 3)

(a) Explained variance of principal components (PCs) from principal component analysis (PCA) of metabolic gene expression levels in malignant cells from different tumors in the melanoma dataset. Top PCs accounting for 80% of the variance are highlighted in red.

(b) Same as in (a) but for the HNSCC dataset.

Supplementary Figure 10

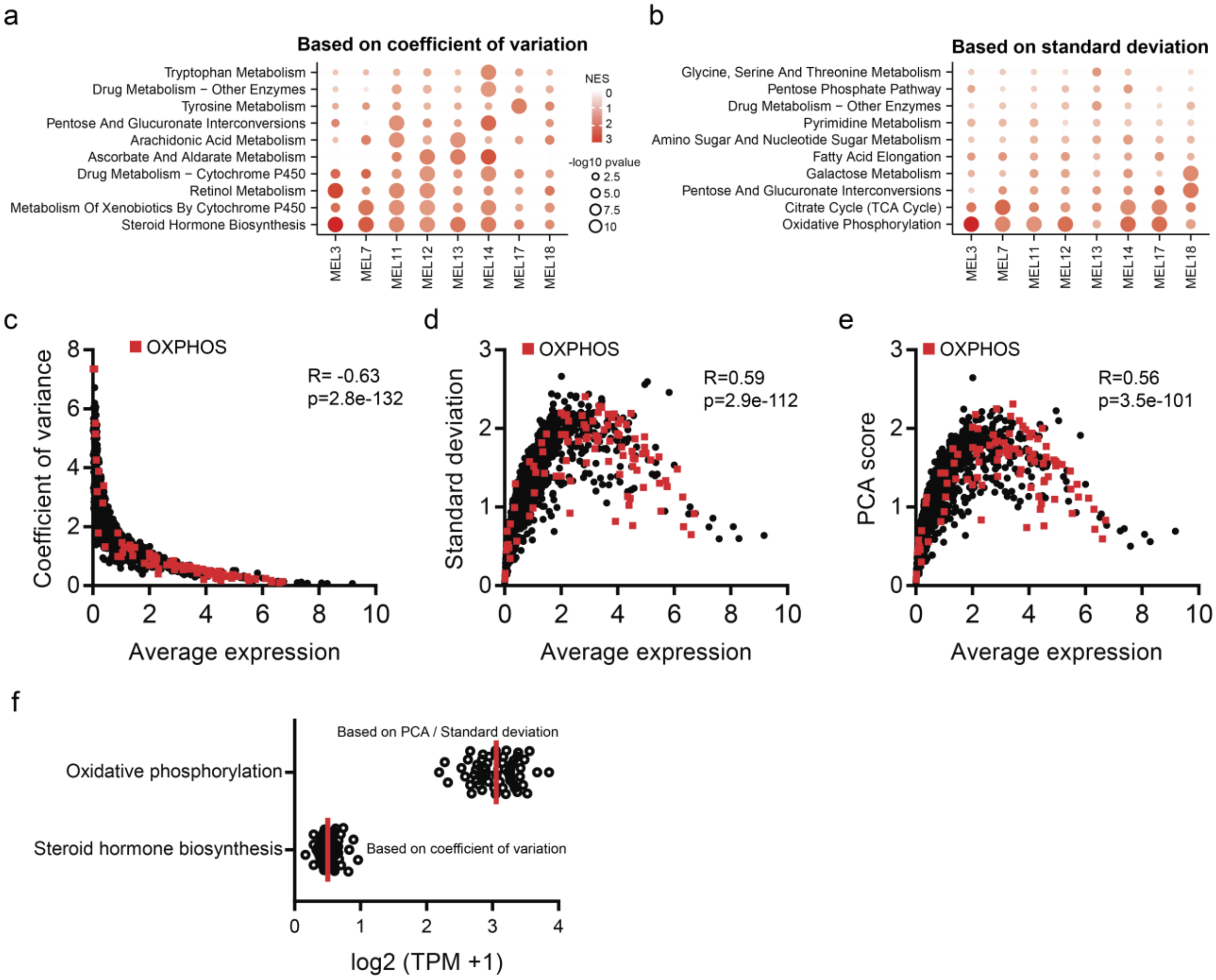

Supplementary Figure 10. (Related to Figure 3)

(a) Metabolic pathways enriched in genes with highest coefficient of variation (CV) in melanoma dataset.

(b) Same as in (a) but for genes with highest standard deviation (SD).

(c) Scatter plot comparing mean gene expression and CV in malignant cells from the melanoma tumor MEL7 as a representative example.

(d) Same as in (c) but for SD.

(e) Same as in (c) but for PCA score.

(f) Swarm plots comparing distributions of gene expression levels in OXPHOS and steroid hormone biosynthesis. Red lines indicate mean values.

Supplementary Figure 11

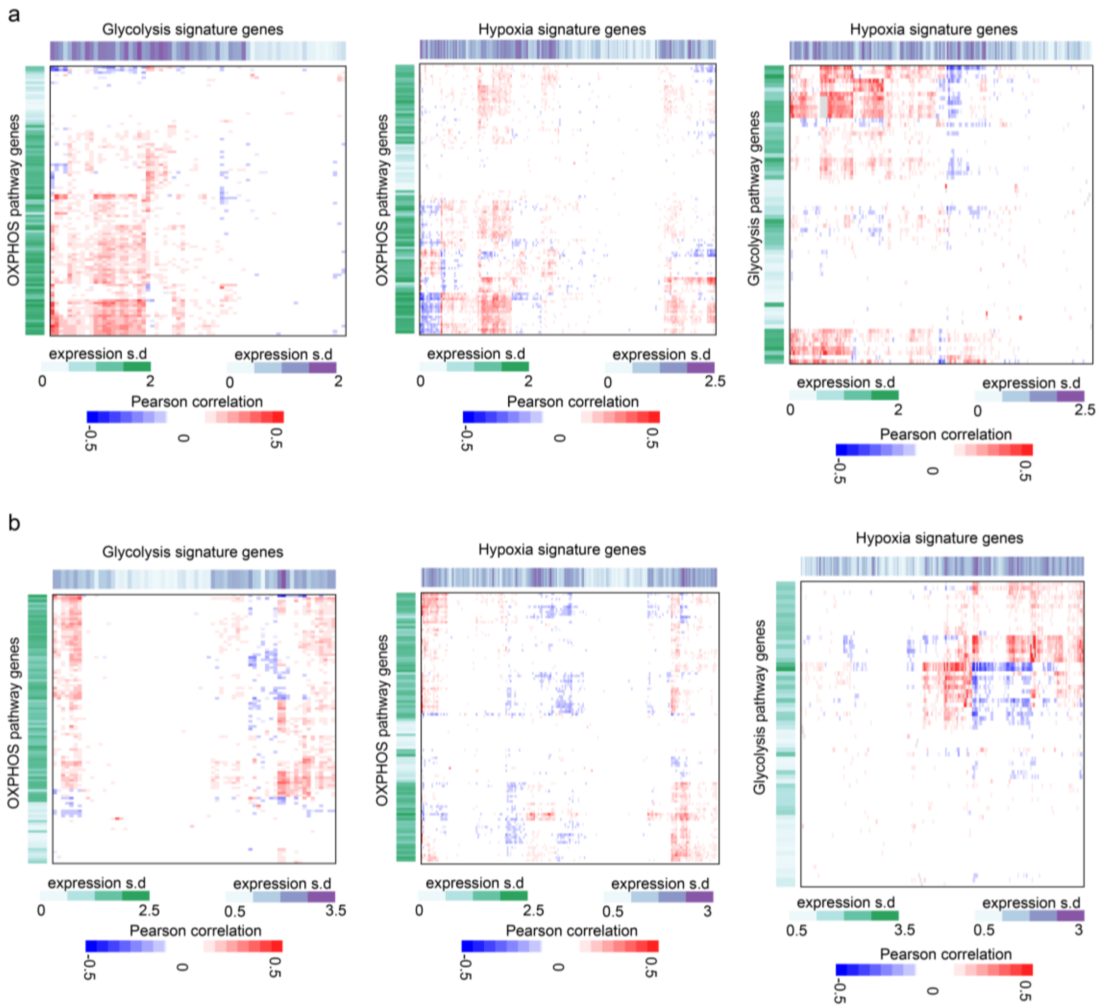

Supplementary Figure 11. (Related to Figure 3)

(a) Pairwise Pearson's correlation coefficients of gene expression levels between oxidative phosphorylation (OXPHOS), glycolysis, and response to hypoxia in malignant cells from the melanoma dataset. Color bars on the left and top show the standard deviations of expression levels of the genes.

(b) Same as in (a) but for HNSCC dataset.

Supplementary Figure 12

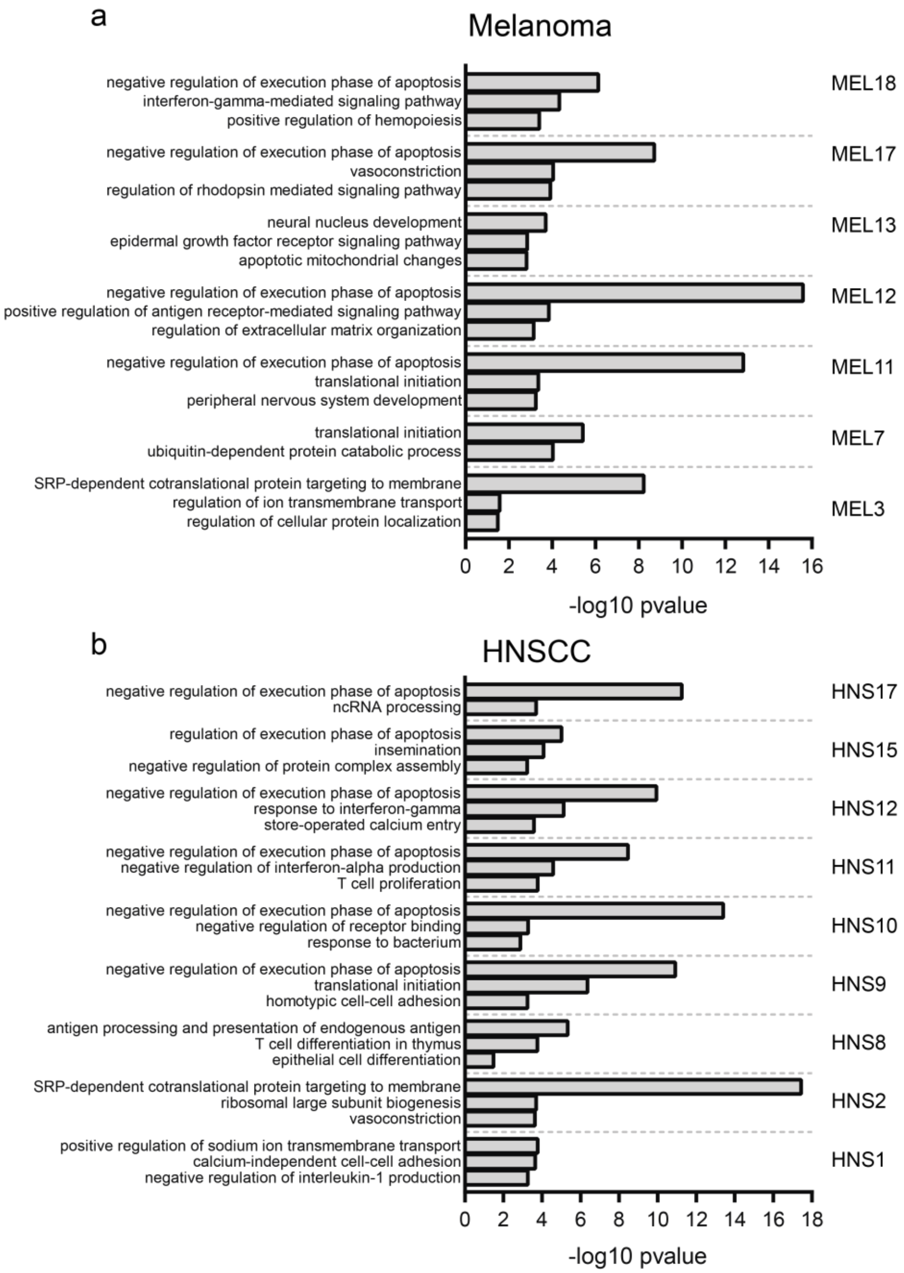

Supplementary Figure 12. (Related to Figure 3)

(a) Top3 GO terms enriched in genes up-regulated in cells with lowest expression levels of glycolysis, OXPHOS and hypoxia pathways for each patient in the melanoma dataset.

(b) Same as in (a) but for HNSCC dataset.

Supplementary Figure 13

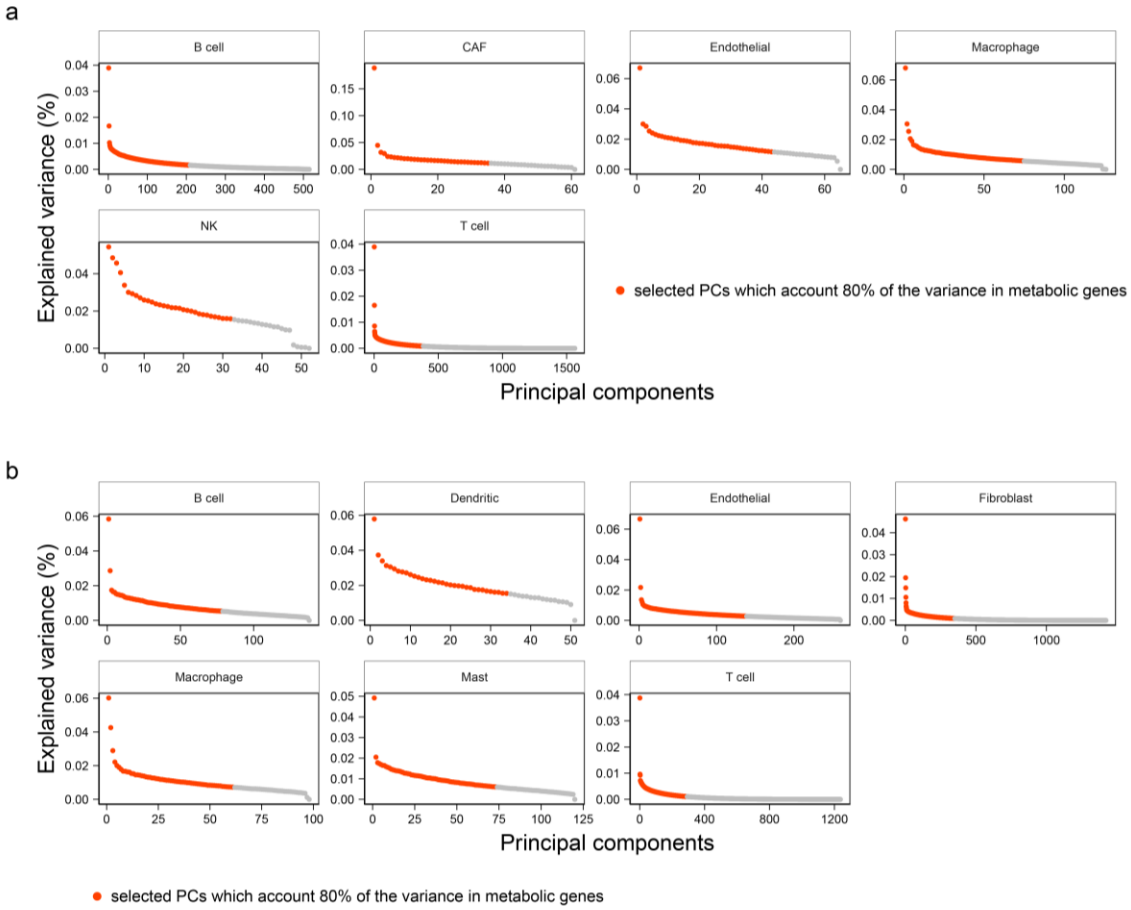

Supplementary Figure 13. (Related to Figure 4)

(a) Explained variance of principal components (PCs) from principal component analysis (PCA) of metabolic gene expression levels in different types of non-malignant cells from the melanoma dataset. Top PCs accounting for 80% of the variance are highlighted in red.

(b) Same as (a) but for HNSCC dataset.

Supplementary Figure 14

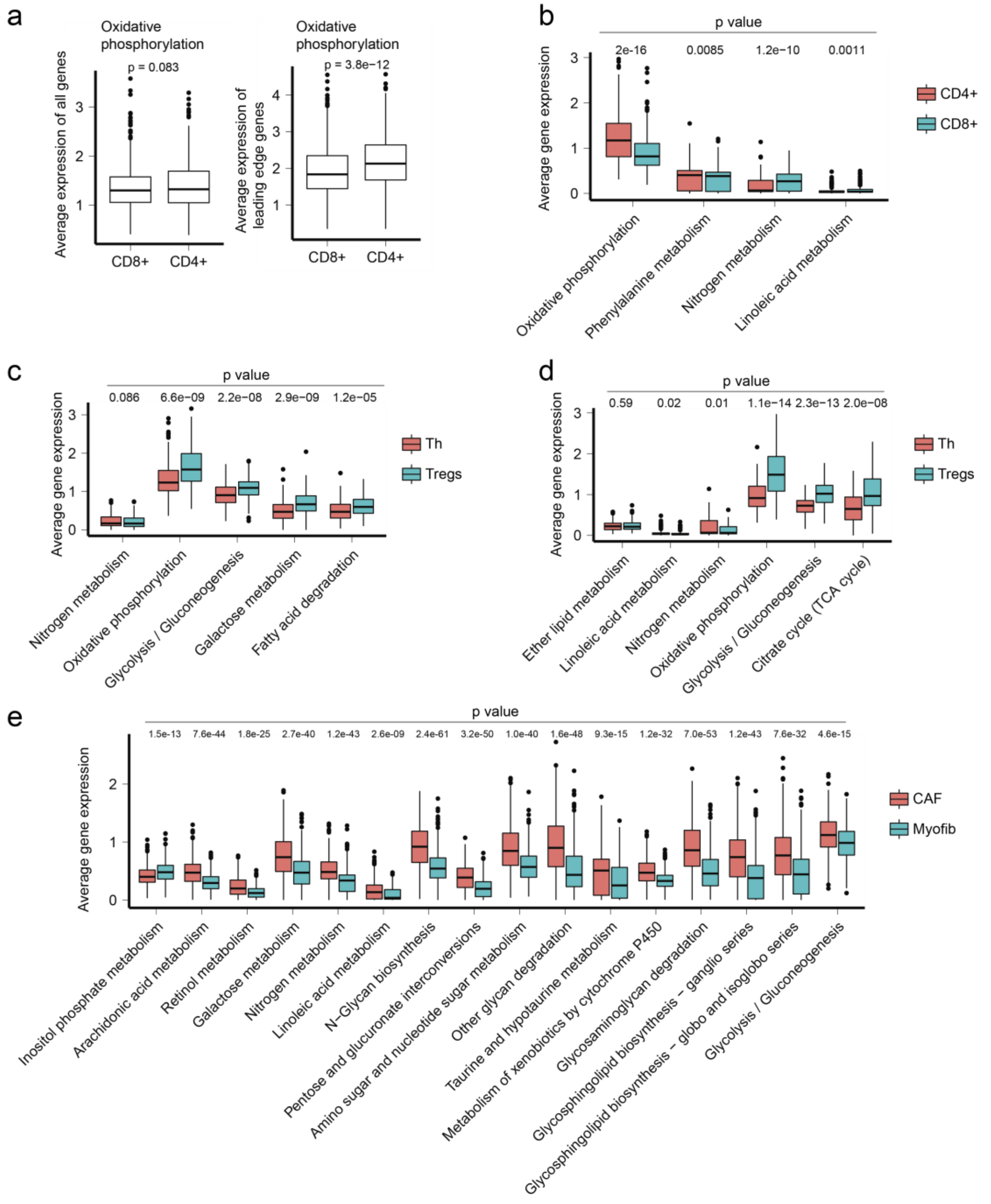

Supplementary Figure 14. (Related to Figure 5)

(a) Distributions of average expression of all OXPHOS genes (left) and the leading-edge genes enriched in the GSEA analysis (right) in CD4+ and CD8+ T cells in the melanoma dataset.

(b) Distributions of average gene expression levels for pathways differentially expressed between CD4+ and CD8+ T cells in the HNSCC dataset.

(c) Same as in (b) but for pathways differentially expressed in Th and Treg cells in the melanoma dataset.

(d) Same as in (c) but for the HNSCC dataset.

(e) same as in (d) but for pathways differentially expressed in CAF cells and Myofib cells in the HNSCC dataset.

P-values shown in each graph were computed using one-sided Wilcoxon's rank-sum test for comparison of gene expression levels between the cell subtypes. Box-plot elements: box limits, 25 and 75 percentiles; center line, median; whiskers, 1.5 × interquartile range (IQR); points, outliers.

Supplementary Table 1. Relative mutual information between t-SNE results generated using different gene sets

**Data set**		**Metabolic gene set *vs* Complete gene set**	**Metabolic gene set *vs* Random gene set**
Melanoma	Malignant cells	1	1
	Non-malignant cells	0.96	0.96
HNSCC	Malignant cells	1	1
	Non-malignant cells	1	1
