# Supplementary material for: Metabolic landscape of the tumor microenvironment at single cell resolution
Source: Nat Commun. 2019 Aug 21;10:3763. doi: 10.1038/s41467-019-11738-0 (PMC6704063; doi:10.1038/s41467-019-11738-0)
Supplement: Supplementary file 5 — Description of Additional Supplementary Files [file 41467_2019_11738_MOESM5_ESM.pdf]

Title: Supplementary Data 1.
Description: Pathway activity scores and related p-values in different cell types”

Title: Supplementary Data 2.
Description: Complete results of the GSEA analysis for comparison of metabolic gene expression between T cell and fibroblast subtypes.

BOX DUMC 3813, Durham, NC 27710 TEL 919.684.5224 EMAIL jason.locasale@duke.edu
LOC C270 LSRC, 450 Research Drive
Durham, NC 27710
